# Supplementary material for: Enantioselective Recognition of Lysine and Phenylalanine Using an Imidazole Salt-Type Fluorescent Probe Based on H8-BINOL
Source: Molecules. 2022 Dec 2;27(23):8470. doi: 10.3390/molecules27238470 (PMC9739330; doi:10.3390/molecules27238470)
Supplement: Supplementary file 1 [file molecules-27-08470-s001.zip › molecules-2018437-supplementary.pdf]

Supplementary materials

Enantioselective recognition of lysine and phenylalanine by an imidazole salt-type fluorescent probe based on H_8-BINOL

Zhaoqin Wei[1], Shi Tang[1], Xiaoxia Sun*[,1] and Yu Hu*[,2]

[1]Jiangxi Key Laboratory of Organic Chemistry, Jiangxi Science and Technology Normal University, Nanchang 330013, China

[2]College of Chemistry, Nanchang University, Nanchang 330031, China

Email: sunxiaoxia77@126.com (X. Sun). huyu@ncu.edu.cn (Y. Hu)

Contents

Supplementary materials.......................................1
二、^{1}H NMR, ^{13}C NMR and MS-ESI.......................................3
2.1 MS-ESI of (*R*)-1.......................................9
2.1 MS-ESI of (*S*)-1.......................................9
三、Figure S1.......................................10
四、Fluorescence experiments of (*S*)-1 for lysine and phenylalanine.......................................10

二、^{1}H NMR, ^{13}C NMR and MS-ESI

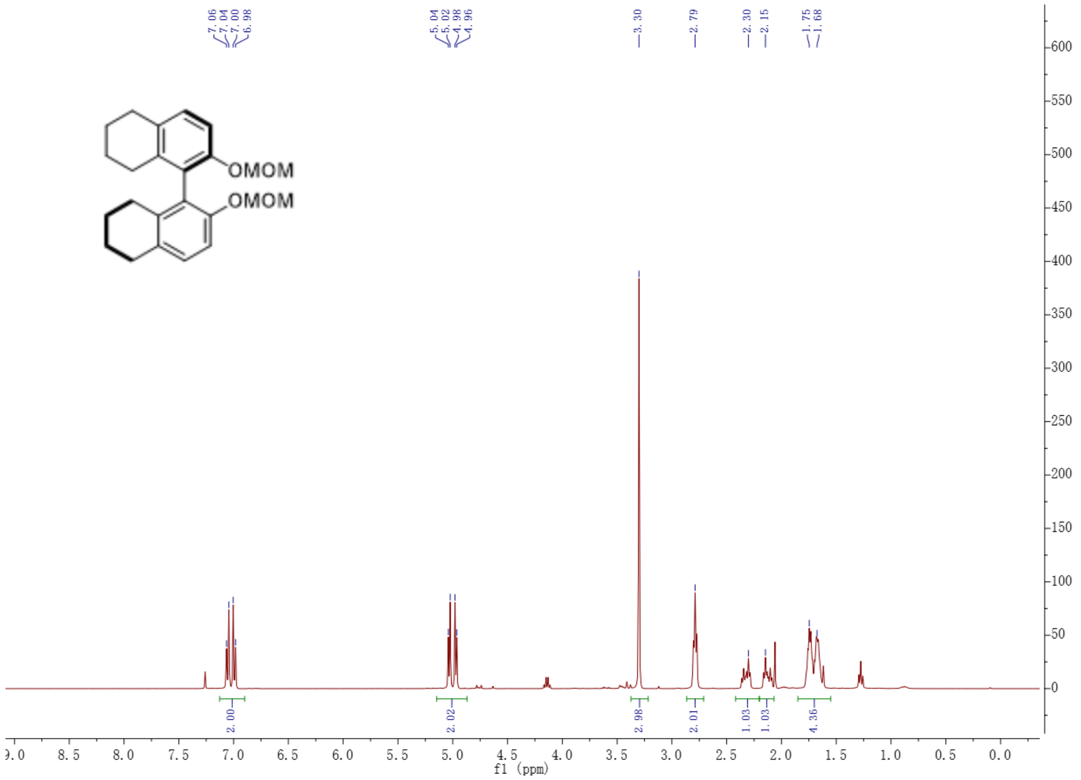

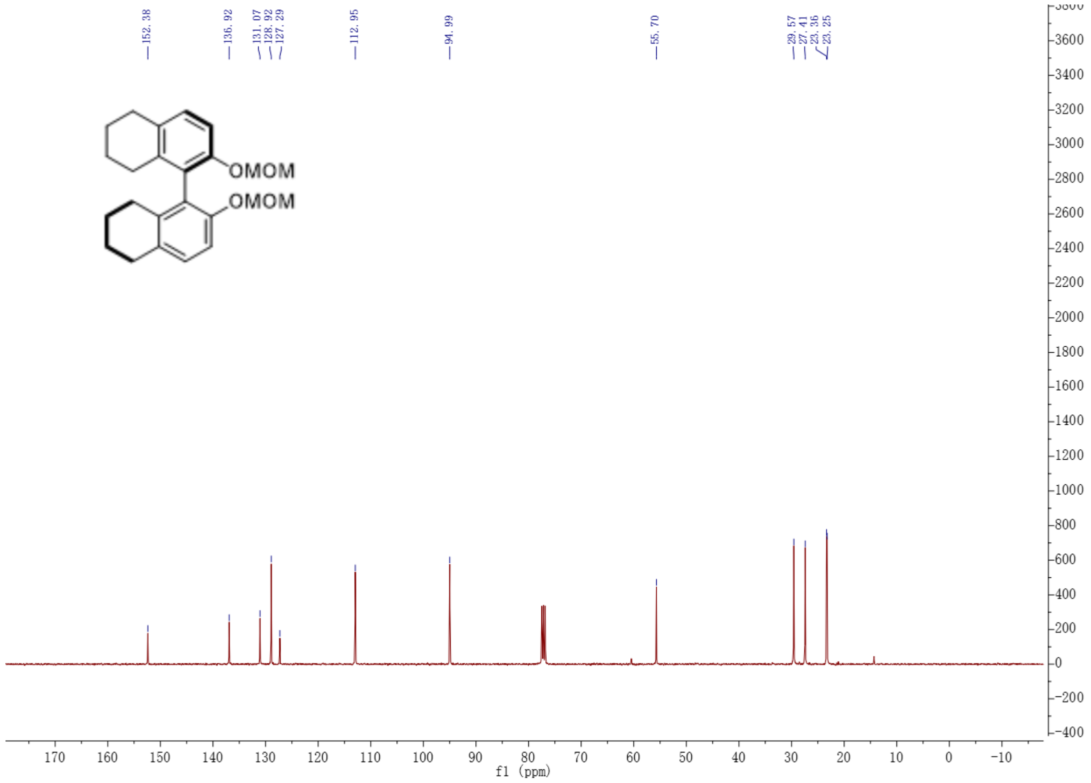

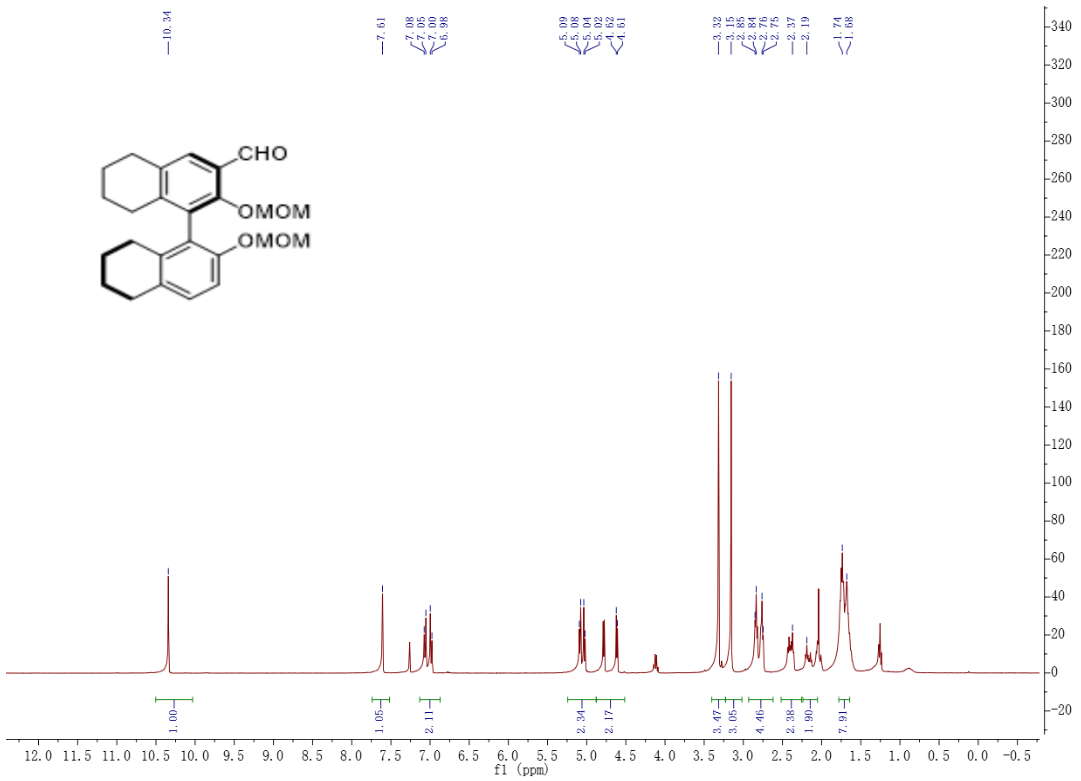
CHO
OMOM
OMOM
10.34
7.61
7.08
7.05
7.00
6.98
5.09
5.08
5.04
5.02
4.62
4.61
3.32
3.15
2.85
2.84
2.76
2.75
2.37
2.19
1.74
1.68
1.00
1.05
2.11
2.34
2.17
3.47
3.05
4.46
2.38
1.90
7.91
f1 (ppm)

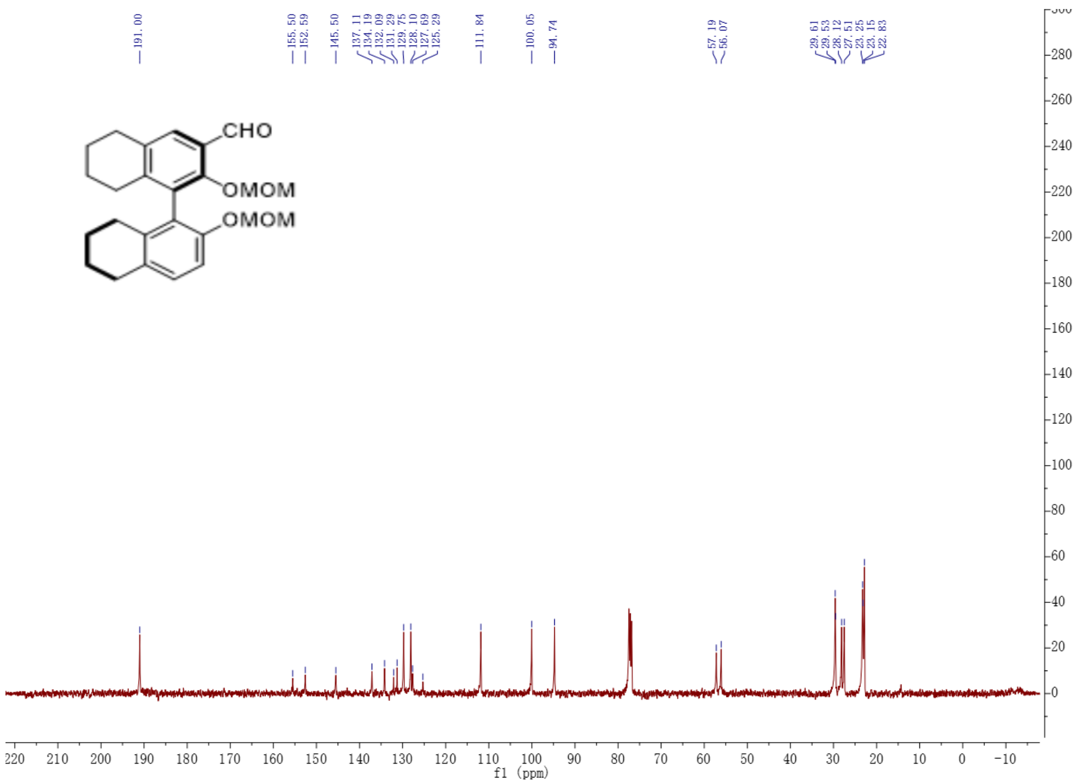
CHO
OMOM
OMOM
191.00
155.50
152.59
145.50
137.11
134.19
132.09
131.29
129.75
128.10
127.69
125.29
111.84
100.05
94.74
57.19
56.07
29.61
29.53
28.12
27.51
23.25
23.15
22.83
f1 (ppm)

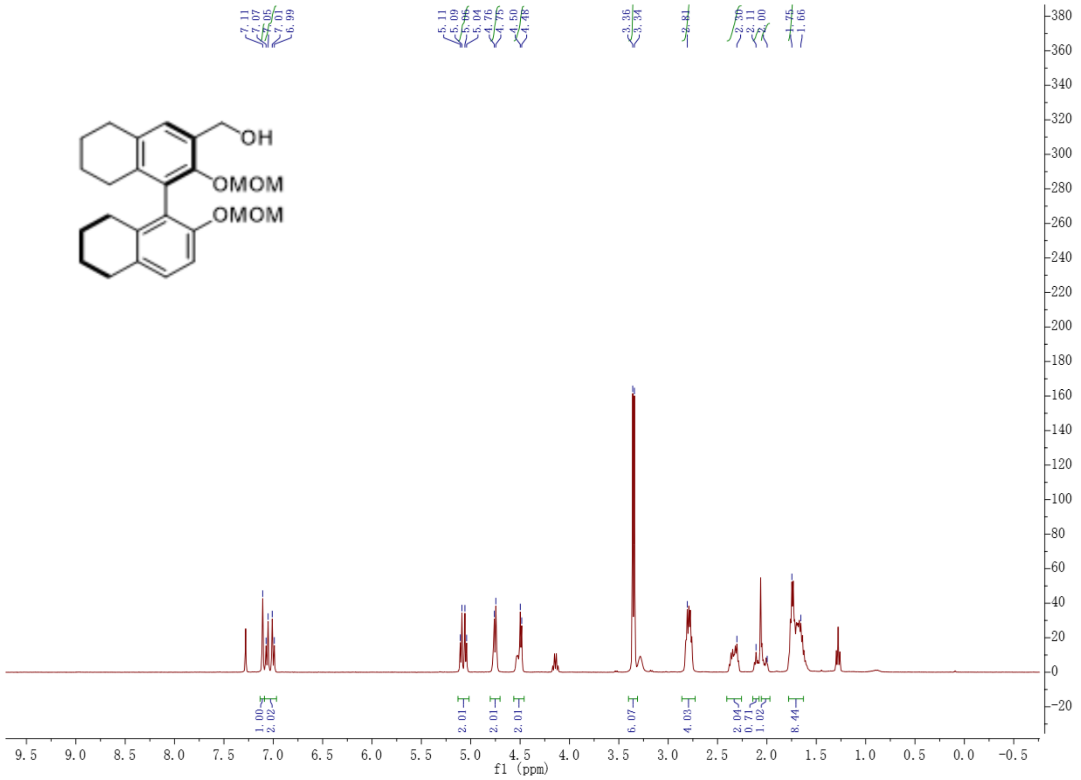

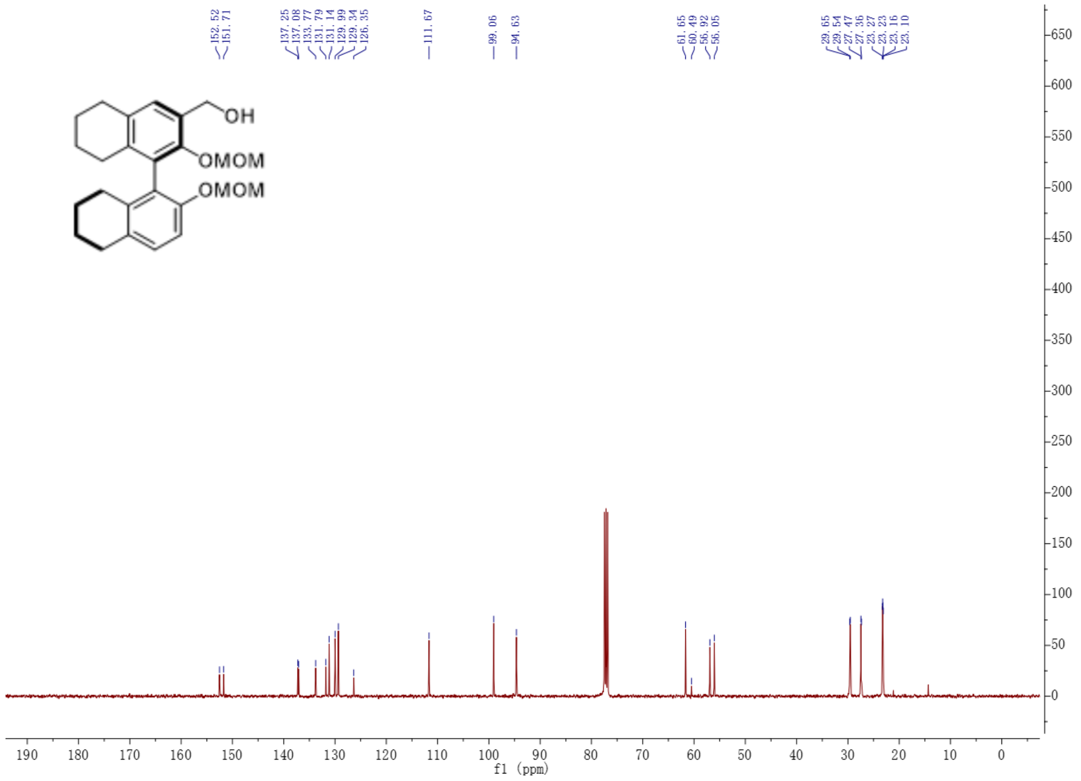

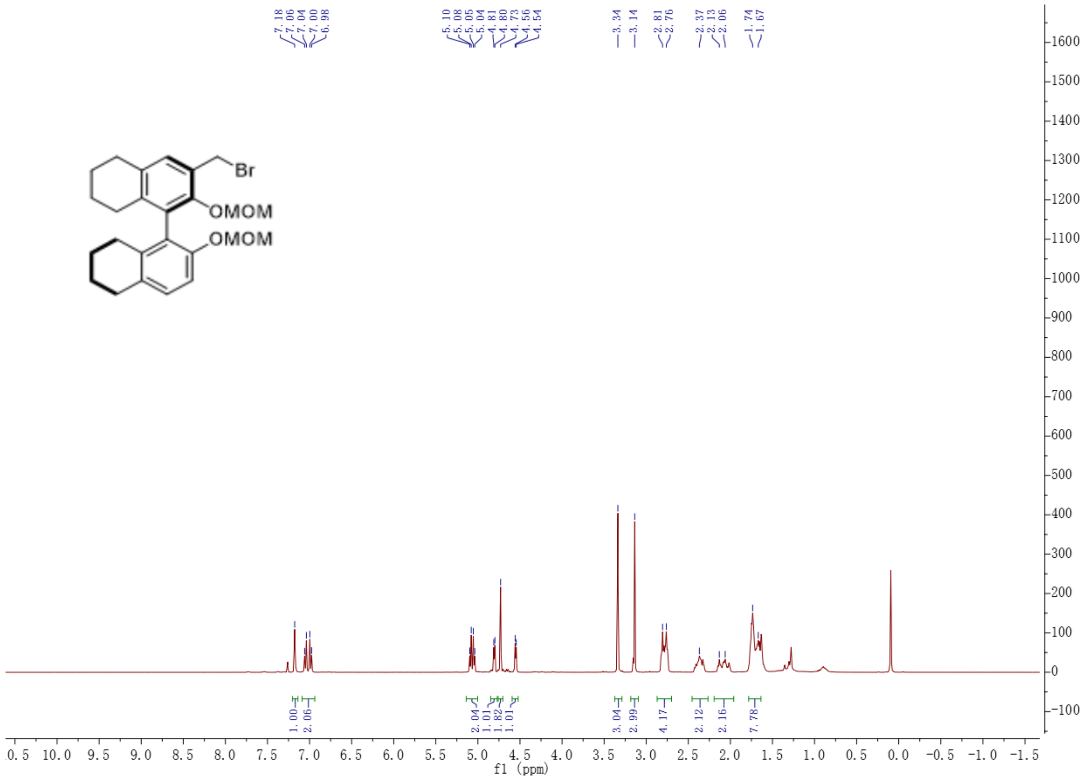
7.18
7.06
7.04
7.00
6.98
5.10
5.08
5.05
5.04
4.81
4.80
4.73
4.56
4.54
3.34
3.14
2.81
2.76
2.37
2.13
2.06
1.74
1.67
Br
OMOM
OMOM
1.00
2.06
2.04
1.01
1.82
1.01
3.04
2.99
4.17
2.12
2.16
7.78
f1 (ppm)

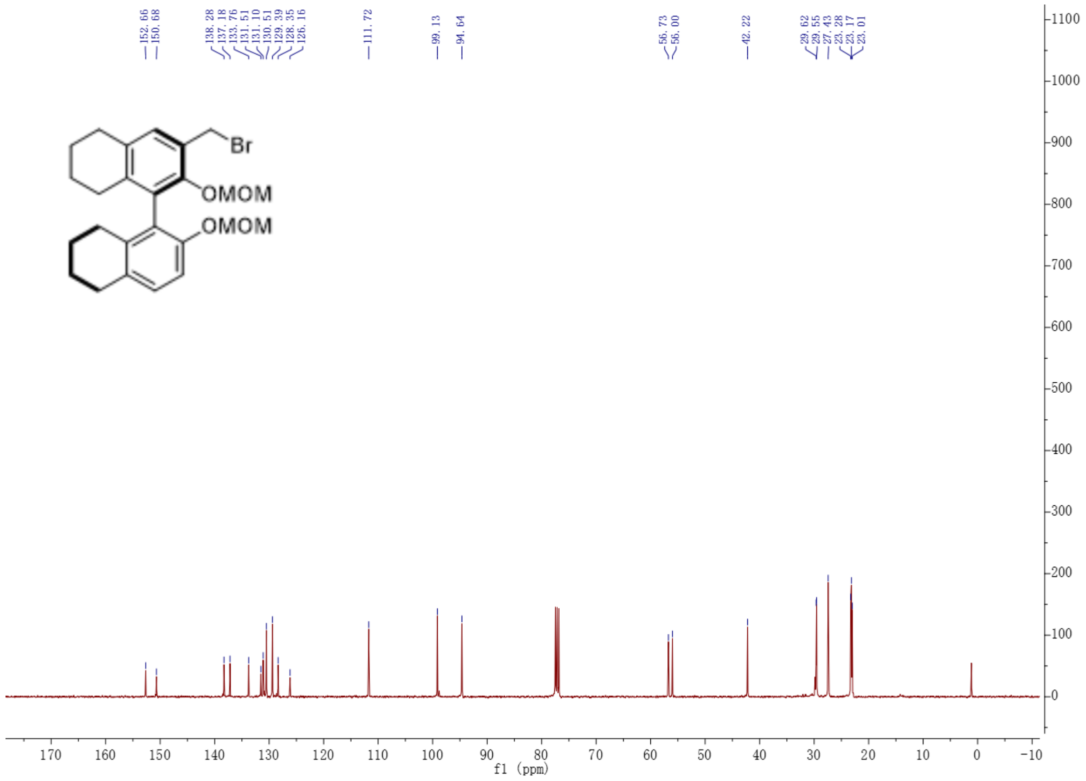
152.66
150.68
138.28
137.18
133.76
131.51
131.10
130.51
129.39
128.35
126.16
111.72
99.13
94.64
56.73
56.00
42.22
29.62
29.55
27.43
23.28
23.17
23.01
Br
OMOM
OMOM
f1 (ppm)

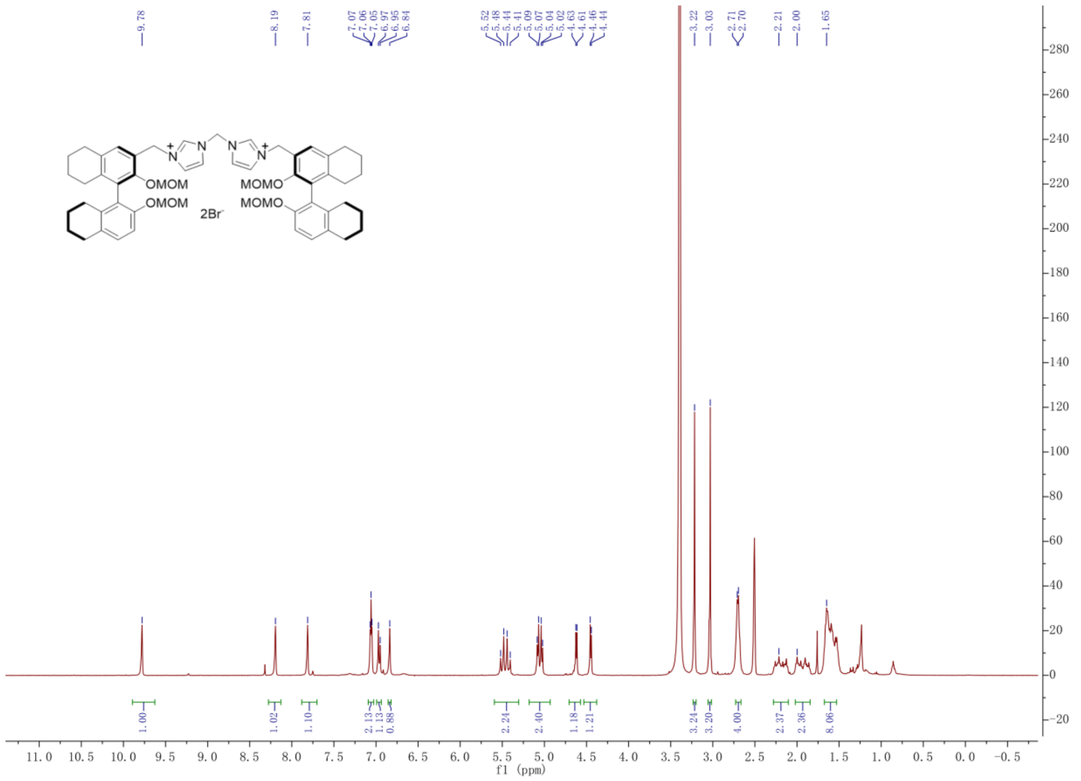

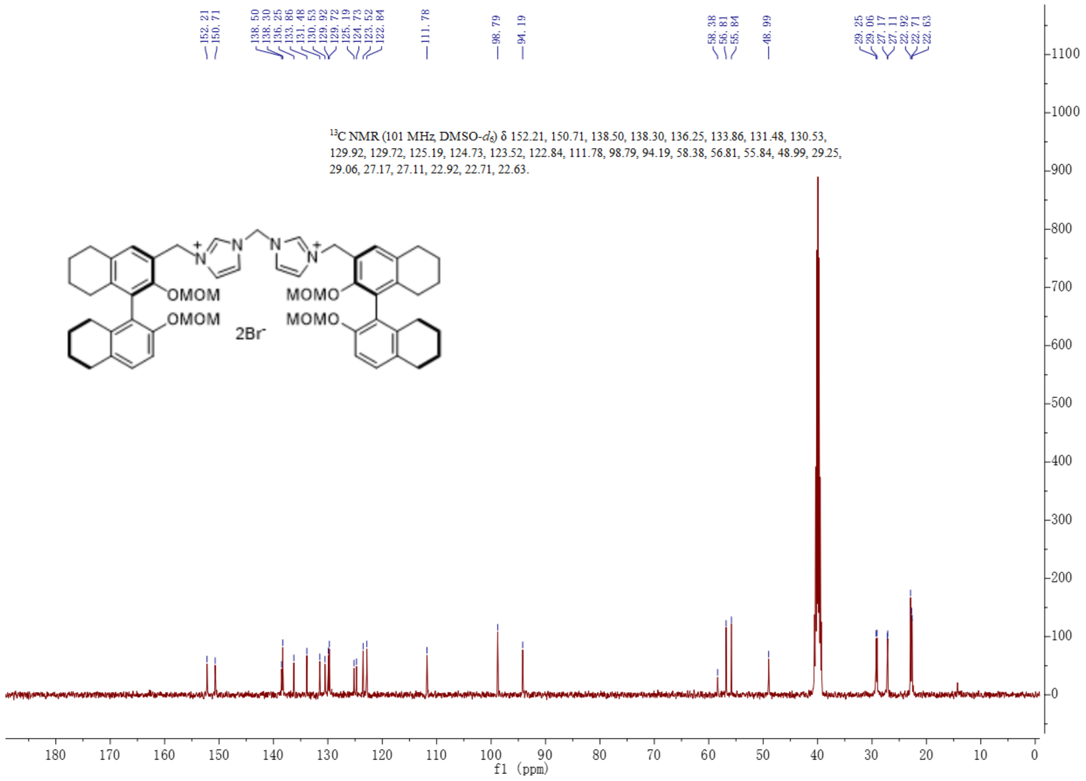

^{13}C NMR (101 MHz, DMSO-d_6) δ 152.21, 150.71, 138.50, 138.30, 136.25, 133.86, 131.48, 130.53, 129.92, 129.72, 125.19, 124.73, 123.52, 122.84, 111.78, 98.79, 94.19, 58.38, 56.81, 55.84, 48.99, 29.25, 29.06, 27.17, 27.11, 22.92, 22.71, 22.63.

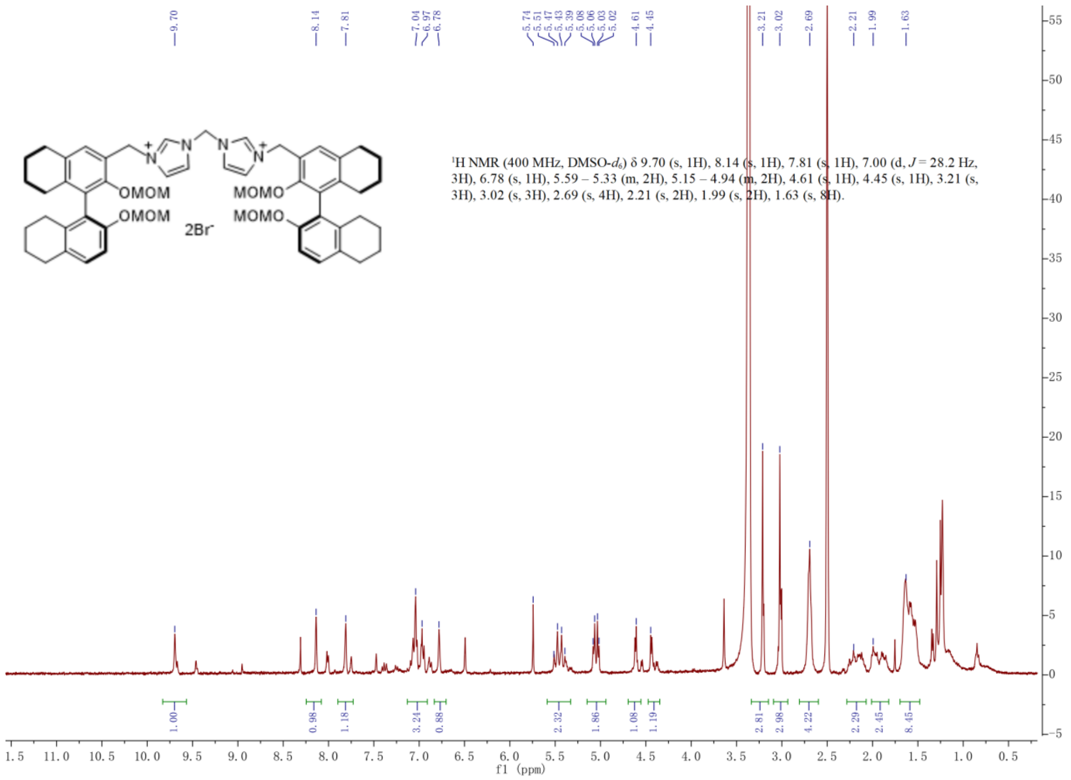

OMOM
OMOM
MOMO
MOMO
2Br⁻
¹H NMR (400 MHz, DMSO-d6) δ 9.70 (s, 1H), 8.14 (s, 1H), 7.81 (s, 1H), 7.00 (d, J = 28.2 Hz, 3H), 6.78 (s, 1H), 5.59 – 5.33 (m, 2H), 5.15 – 4.94 (m, 2H), 4.61 (s, 1H), 4.45 (s, 1H), 3.21 (s, 3H), 3.02 (s, 3H), 2.69 (s, 4H), 2.21 (s, 2H), 1.99 (s, 2H), 1.63 (s, 8H).
f1 (ppm)

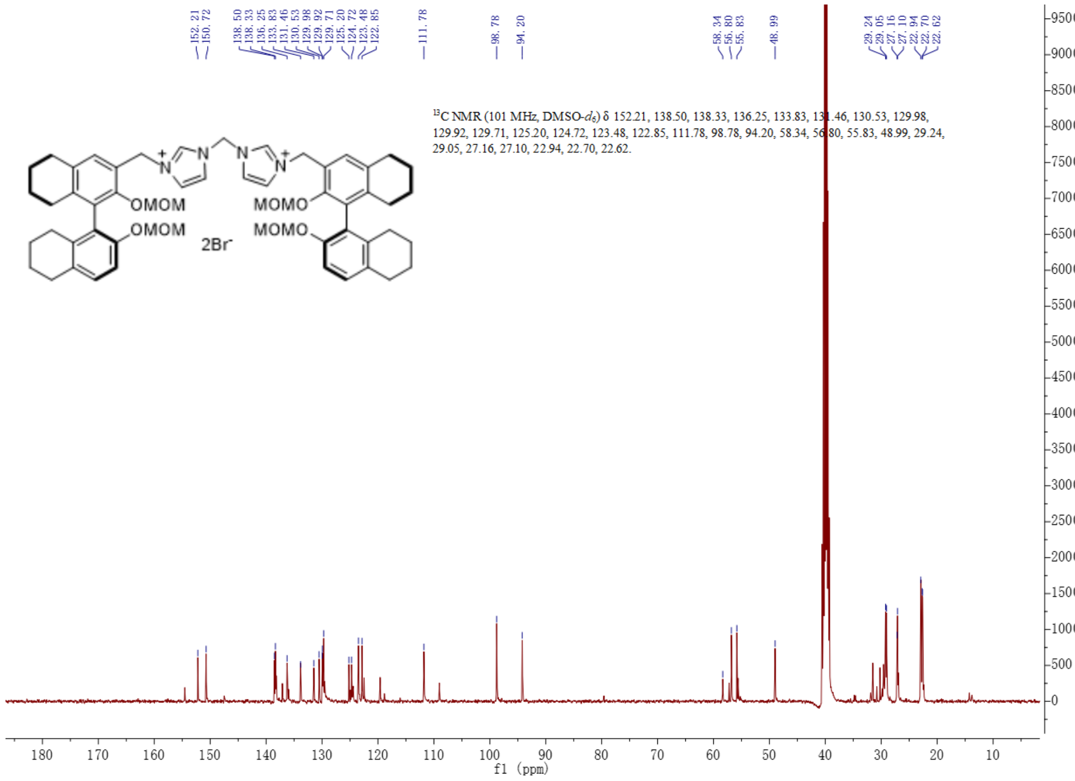

OMOM
OMOM
MOMO
MOMO
2Br⁻
¹³C NMR (101 MHz, DMSO-d6) δ 152.21, 138.50, 138.33, 136.25, 133.83, 131.46, 130.53, 129.98, 129.92, 129.71, 125.20, 124.72, 123.48, 122.85, 111.78, 98.78, 94.20, 58.34, 56.80, 55.83, 48.99, 29.24, 29.05, 27.16, 27.10, 22.94, 22.70, 22.62.
f1 (ppm)

2.1 MS-ESI of (*R*)-1

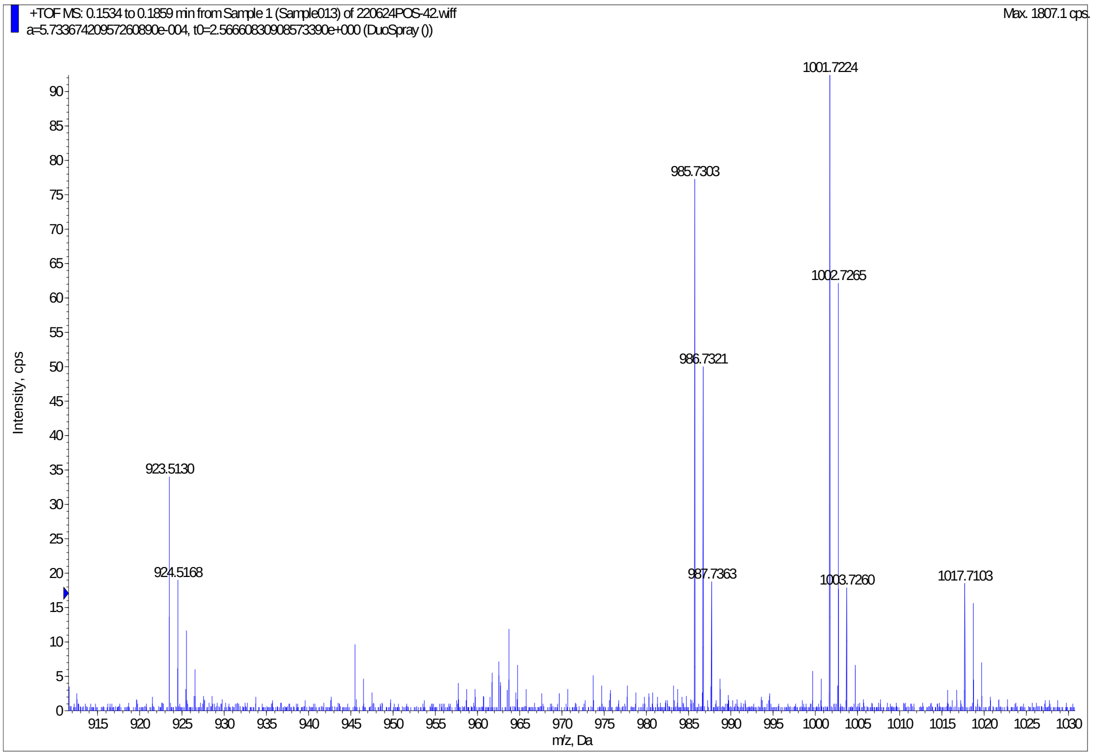

2.1 MS-ESI of (*S*)-1

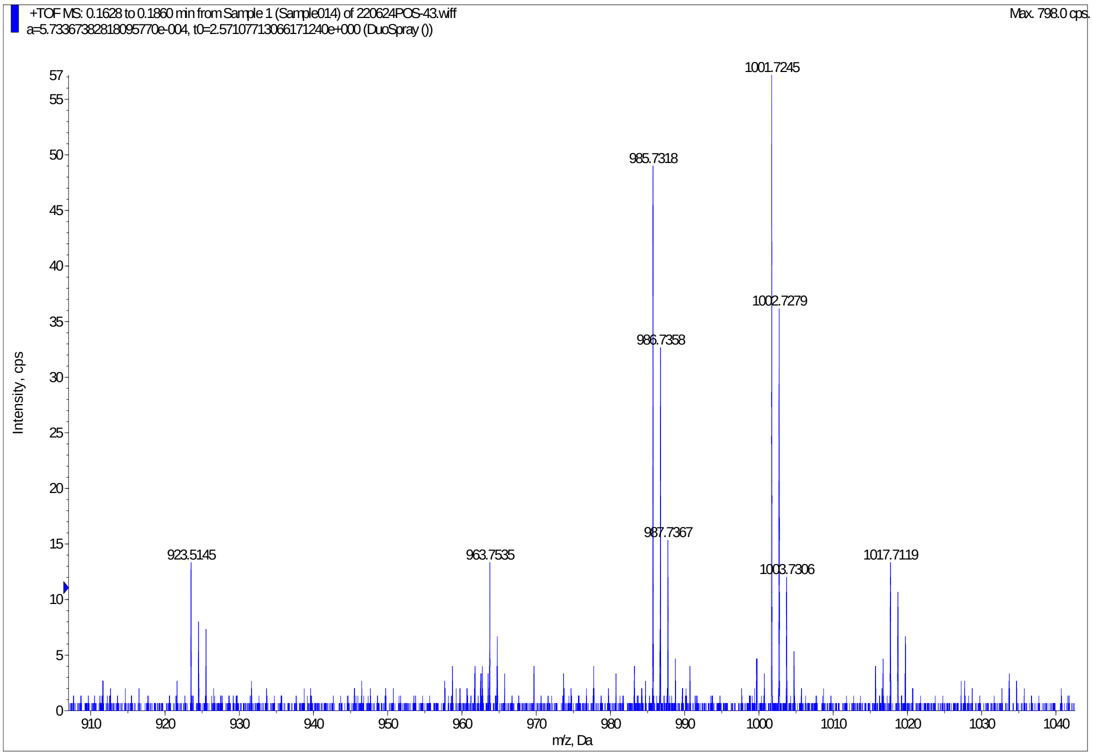

三、Figure S1

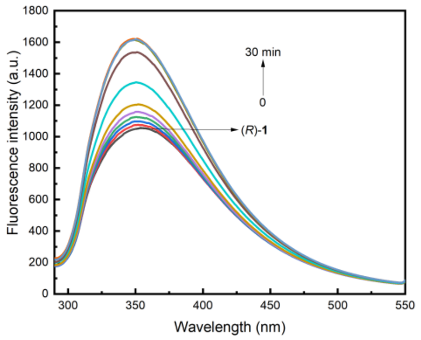

Figure S1 Fluorescence intensity change of (*R*)-**1** in an aqueous solution within 30min

四、Fluorescence experiments of (*S*)-**1** for lysine and phenylalanine

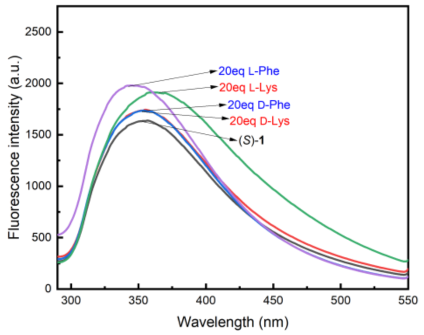

Figure S2 Fluorescence spectra of (*S*)-**1** ($2.0*10^{-5}$ M) with L/D-Lys and L/D-Phe (20.0 equivalents) in aqueous solution. (λexc=280 nm, slits: 5.0/2.5 nm).

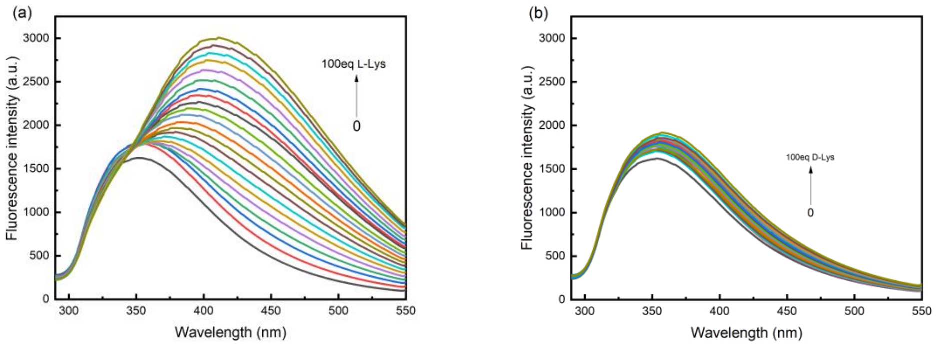

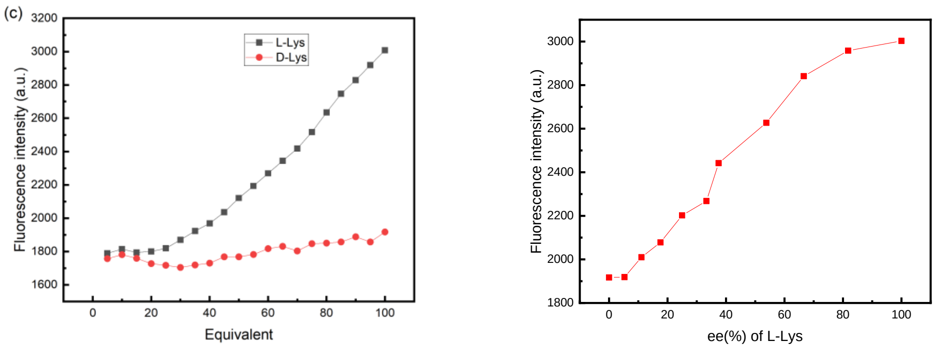

Figure S3 (a) Fluorescence titration spectra of (*S*)-**1** ($2.0*10^{-5}$ M) with L-Lys (0.1M) in aqueous solution. (b) Fluorescence titration spectra of (*S*)-**1** with D-Lys in aqueous solution. (c) Trends of (*S*)-**1** fluorescence intensity after the addition of different equivalent Lys. (d) Fluorescence response at λ =349 nm for the mixture of (*S*)-**1** toward L-Lys (100.0 eq) at various ee (λexc=280 nm, slits: 5.0/2.5 nm).

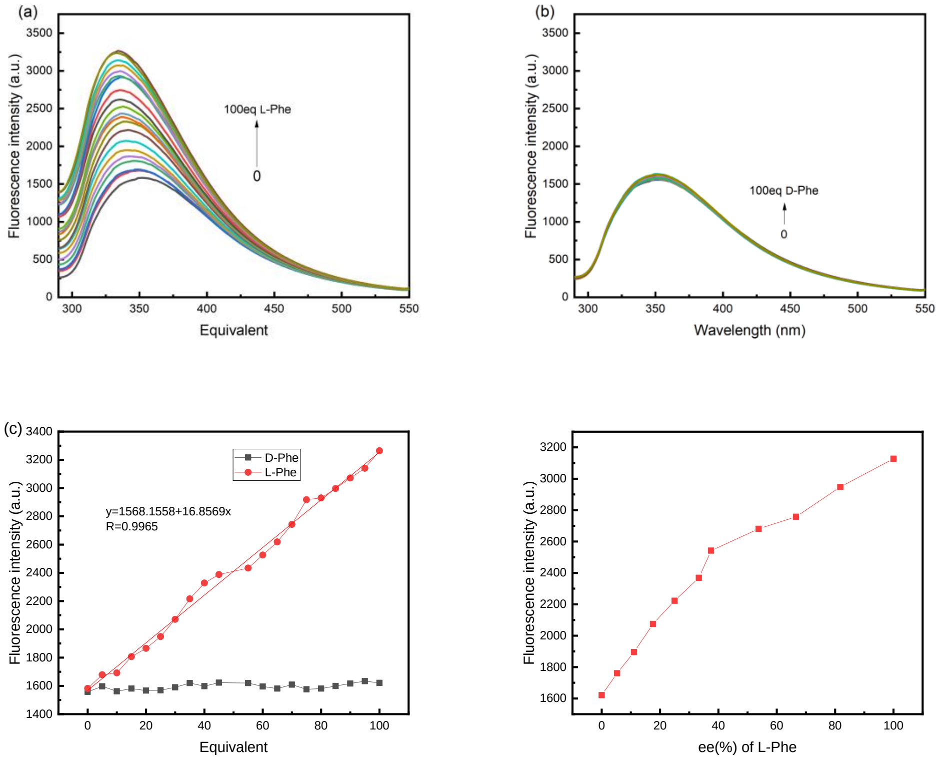

Figure S4 (a) Fluorescence titration spectra of (*S*)-**1** ($2.0*10^{-5}$ M) with L-Phe (0.1M) in aqueous solution. (b) Fluorescence titration spectra of (*S*)-**1** with D-Phe in aqueous solution. (c) Trends of (*S*)-**1** fluorescence intensity after the addition of different equivalent Phe. (d) Fluorescence response at λ =349 nm for the mixtures of (*S*)-**1** toward L-Phe (100.0 eq) at various ee. (λexc=280 nm, slits: 5.0/2.5 nm)
